# Sex differences in the physiological response to acute anterior cruciate ligament overuse

**DOI:** 10.1113/JP289078

**Published:** 2025-10-29

**Authors:** Stephen H. Schlecht, Benjamin E. Loflin, Adam R. Carter, Roufael Hanna, Simran Shergill, Edward M. Wojtys

**Affiliations:** ^1^ Department of Orthopaedic Surgery Indiana University School of Medicine Indianapolis IN USA; ^2^ Department of Anatomy, Cell Biology and Physiology Indiana University School of Medicine Indianapolis IN USA; ^3^ Department of Kinesiology Indiana University – Indianapolis Indianapolis IN USA; ^4^ Department of Biology Indiana University – Indianapolis Indianapolis IN USA; ^5^ Department of Orthopaedic Surgery University of Michigan Ann Arbor MI USA

**Keywords:** anterior cruciate ligament, fatigue loading, mouse model, sex differences, tissue overuse

## Abstract

**Abstract:**

Young female athletes are at least two times more likely to suffer a non‐contact anterior cruciate ligament (ACL) injury than males, and one and a half times more likely to have a recurrent injury. Primary factors contributing to this disparity are less stiff and weaker ACLs, and greater knee laxity than males. Also, some evidence suggests females may exhibit a muted response to repetitive, high‐intensity activity compared to males. Here, we test the hypothesis that female ACLs would accumulate more extracellular matrix (ECM) damage and show a delayed reparative response compared to males under equivalent submaximal fatigue loading. Using an adolescent mouse model (C57BL/6J), ACLs were cyclically loaded to induce an acute submaximal overuse injury (*n* = 20 per sex). ECM damage was assessed via immunofluorescence, apoptotic activity via immunohistochemistry, and gene expression changes through RNA‐sequencing at 24 and 72 h post‐injury. Female ACLs showed significantly greater collagen denaturation than males (*P* = 0.05), with no significant difference in apoptosis. Transcriptomic analyses suggest sex‐specific healing strategies. Females followed a slower, more regulated reparative response, whereas males exhibited a more aggressive repair approach emphasizing mitosis, cell proliferation and migration. These findings may explain higher female ACL failure rates as greater matrix damage combined with a slower repair response could lead to injury propagation if reloading occurs prematurely. By contrast, the faster male response might reduce recurrence risk but increase fibrosis potential. If confirmed further, these potential physiological differences may require the implementation of sex‐specific strategies for training and recovery regimens to prevent overuse injuries and optimize healing outcomes in young athletes.

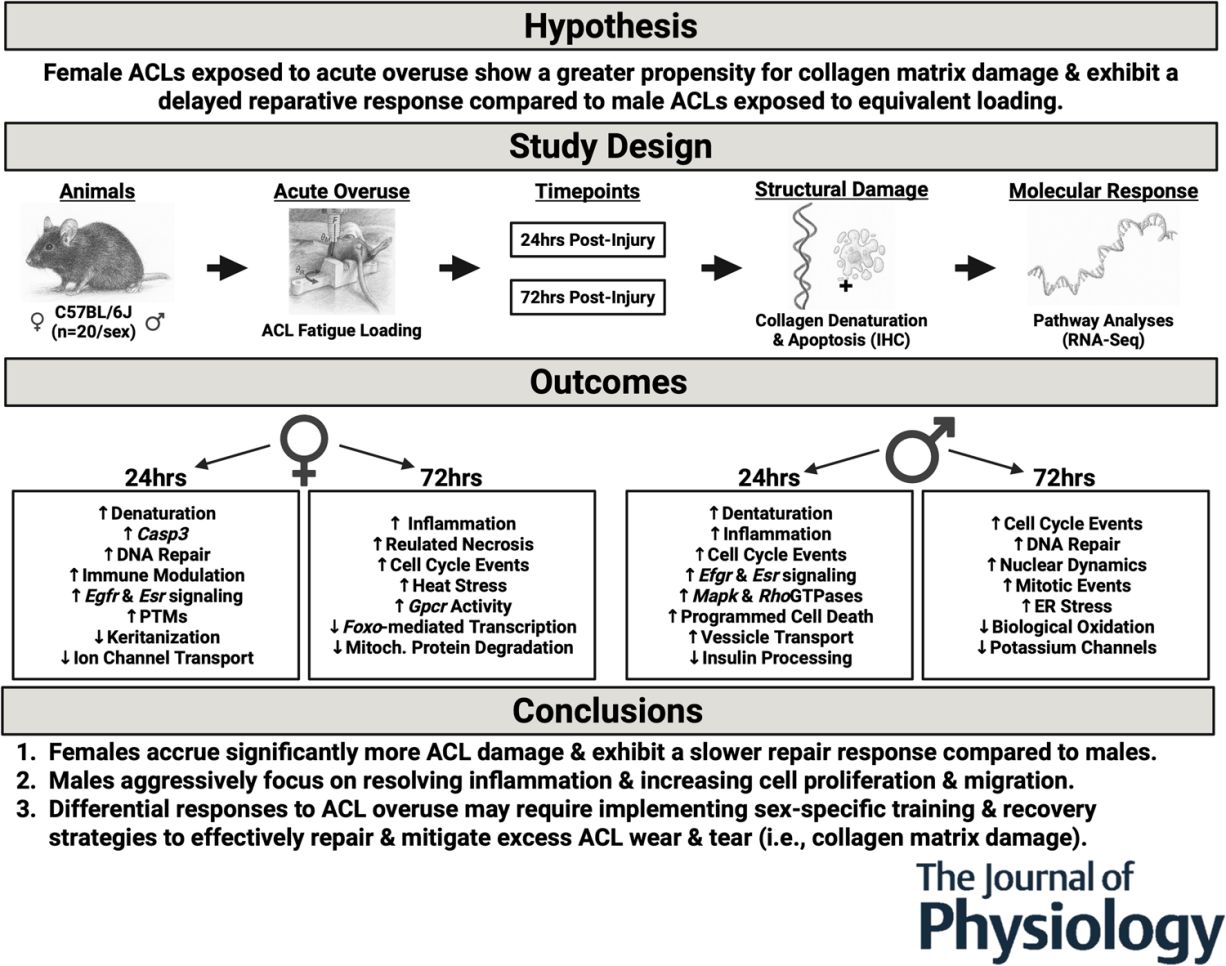

**Key points:**

- Females are at least twice as likely to suffer an anterior cruciate ligament injury (ACL) relative to males when participating in the same, or comparable sport.
- Sex differences in ACL structure and function are primary factors for increased female injury risk.
- Here, we show that the female mouse ACL accrues more collagen matrix damage than males under comparable fatigue loads.
- Additionally, female mice demonstrated a slower, more regulated ACL reparative response, while male mice exhibited a more aggressive repair approach emphasizing mitosis, cell proliferation and migration.
- These results, if translatable, may in part explain the sex‐disparity in ACL failure rates with greater matrix damage and a slower repair response increasing the risk of reinjury, and a faster male response potentially reducing injury recurrence risk with further physical activity.

## Graphical abstract

**Abstract figure legend** With equivalent *in vivo* fatigue loading of the anterior cruciate ligament (ACL), females accumulate more tissue damage and mount a slower reparative response than males, when the applied loads between sexes are equivalent. By contrast, males accumulate less tissue damage and mount a robust reparative response that includes resolving inflammation and promoting cell proliferation and migration. With translation, this differential physiological response to ACL overuse may, in part, explain higher non‐contact injury rates in females.

## Introduction

Anterior cruciate ligament (ACL) injuries are one of the most common orthopaedic injuries in the USA, with at least 200,000 cases annually (Sanders et al., 2016). More than half of ACL injuries occur in individuals between 15 and 25 years of age (Griffin et al., 2006) and primarily result from non‐contact mechanisms (Boden et al., 2000). Among high school and collegiate athletes, females are at least two times more likely to suffer a first‐time non‐contact ACL failure than males when accounting for sport played (Beynnon et al., 2014) and at least one and a half times more likely to suffer a recurrent ACL injury (Slater et al., 2019). ACL injuries can be debilitating and increase the likelihood of time away from sport, discontinuation of sport and surgical intervention (Swenson et al., 2013; Welton et al., 2018). Moreover, those that suffer an ACL injury are four to six times more likely to develop knee osteoarthritis (Poulsen et al., 2019), irrespective of receiving conservative or surgical treatment (Friel & Chu, 2013; Roos, 2005).

Reasons for this sex disparity in injury risk are multifactorial and include a suite of anatomical, biomechanical and hormonal differences (Ireland, 2002; Shultz & Fegley, 2023; Shultz et al., 2008). Importantly, females on average have less stiff and weaker ACLs compared to males (Chandrashekar et al., 2006; Lipps et al., 2012), contributing to a greater knee laxity (Shultz et al., 2008). Fluctuations in the female hormonal milieu during the peri‐ovulatory and mid‐luteal days of the menstrual cycle are associated with increased knee laxity and greater anterior tibial translation upon weight acceptance (Herzberg et al., 2017; Shultz et al., 2011; Somerson et al., 2019). Moreover, studies using ovariectomized female animals have shown ACL mechanical properties decrease with oestrogen supplementation, although at supraphysiological levels (Slauterbeck et al., 1999). Furthermore, clinical findings suggest that these soft tissue functional changes arise via sex hormone modulation of collagen metabolism (Shultz et al., 2012). Recent *in vitro* findings from cyclically fatigued rabbit ACLs support this perspective, with female ACLs demonstrating a muted expression of pro‐collagen synthesis genes, but greater upregulation of catabolic genes, whereas male ACLs showed significant upregulation of pro‐collagen genes and little change in catabolic gene expression within the extracellular matrix (ECM) (Paschall et al., 2024). Thus, the collagen remodelling response to loading may be delayed, or muted, in female ACLs, potentially increasing their risk for failure with subsequent physical activity. This is an important finding considering data increasingly suggest that the accumulation of load‐induced ECM damage via repetitive high‐intensity activity probably precedes many non‐contact ACL injuries (Chen et al., 2019; Kim et al., 2022; Loflin et al., 2023; Putera et al., 2023).

Here, we sought to further investigate these sex‐based metabolic differences in the response of the ACL to cyclic loading (i.e. fatigue) *in vivo*, at the same time as determining whether female ACLs demonstrate a greater propensity for collagen matrix damage via acute tissue overuse. We hypothesized that female ACLs would accrue significantly more matrix damage and exhibit a delayed, or prolonged, response to collagen disruptions relative to male ACLs fatigued at equivalent loads. To test this hypothesis, we employed a novel mouse ACL fatigue model to generate an acute overuse injury *in vivo* that we previously showed induces collagen matrix denaturation (i.e. damage) and reduced organ‐level ACL strength and stiffness (Loflin et al., 2023). We then used histological and molecular assays to characterize ECM structural alterations (collagen denaturation, programmed cell death) and the cellular response (differential gene expression) to an acute overuse injury.

## Methods

### Ethical approval

All animal experiments were approved by the Indiana University School of Medicine Institutional Animal Care and Use Committee, which adheres to Association for Assessment and Accreditation of Laboratory Animal Care International guidelines. The institutional approval code for the protocol detailing animal care, experimentation and humane death is No. 22062.

### Experimental design

Male and female C57BL/6J inbred mice were ordered from The Jackson Laboratory at 9 weeks of age (*n* = 20 per sex). Mice were group housed by sex under a 12:12 h light/dark photocycle cycle and provided food and water *ad libitum*. Prior to beginning the study, mice were allowed to acclimate to their new environment for 1 week. At 10 weeks of age, which is an age musculoskeletally comparable to adolescent humans (Dutta & Sengupta, 2016), mice were provided heat support, anesthetized using vaporized isoflurane concentrations appropriate for their weight (5% induction, 1.5–2% maintenance) and had ocular lubrication applied. Anaesthesia depth was monitored via heartrate and paw withdrawal reflex. When sedated, each mouse had their right knee positioned in a custom loading fixture that approximates knee kinematics involved in a jump‐landing and pivot shift to cyclically load the ACL to induce an acute overuse injury. Mice were then recovered from anaesthesia with the provision of heat support, monitored for 1 h and returned to normal cage activity with no analgesia provided. Mouse recovery is monitored every 12 h for up to 3 days following experimentation. Twenty‐four hours after injury, a subset of male and female mice (*n* = 10 per sex) were randomly selected and killed via isoflurane overexposure followed by cervical dislocation for histological and molecular analyses. The remaining mice (*n* = 10 per sex) were killed in the same manner 72 h following injury for the same downstream analyses. Immediately following death, paired hindlimbs were removed from each mouse. The ACLs of four paired hindlimbs were processed for histology. The remaining six paired hindlimbs from each post‐injury timepoint were dissected from the knees and processed for RNA sequencing.

### *In vivo* ACL fatigue loading and knee mechanics

**Figure 1:**
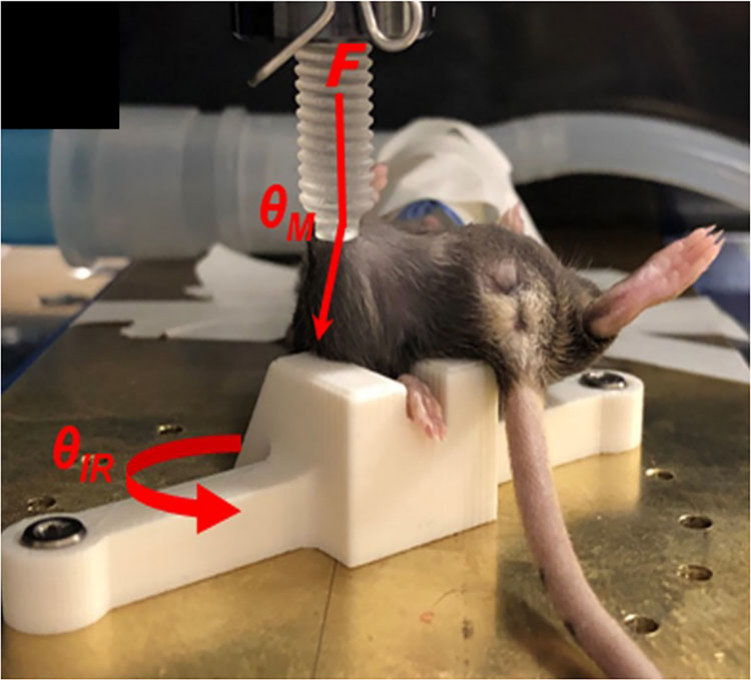
*In vivo* testing setup Knee in valgus, tibia internally rotated. Cyclic (fatigue) or maximal (failure) compressive force applied to femur to tension ACL.

The *in vivo* mouse model accounts for jump‐landing/pivot shift knee kinematics previously employed to characterize the ACL fatigue mechanism *in situ* using cadaveric human knees (Chen et al., 2019; Kim et al., 2022; Putera et al., 2023). For mice, a custom loading jig aligns the knee joint with the testing system actuator at the same time as statically applying a valgus moment across the knee joint and internally rotating the tibia, as previously described (Fig. 1) (Loflin et al., 2023). To fatigue the ACL, mice were provided heat support, anesthetized isoflurane and ocular lubrication. Next, mice were positioned in the loading fixture with a preload of ∼0.25 N applied to the right distal femur to maintain femoral–tibial contact within the knee throughout the loading protocol to prevent ACL trauma confounders (e.g. bone bruising, subchondral fracture) during fatiguing. Right knees were then exposed to interval loading (moderate to strenuous cyclic loading) that mimics load intensity shifts experienced during athletic training and competition, and was previously shown to generate increased *in vivo* collagen matrix denaturation accompanied by a progressive reduction in ACL strength and stiffness (Loflin et al., 2023). The left knees served as internal controls. To define the moderate and strenuous loads prior to testing, a subset of mice (*n* = 10 per sex) had their right ACLs loaded to failure using the exact same fixture and testing protocols as for fatigue loading, except that, instead of cycling the load, the distal femur was axially loaded at a rate of 2.7 N s^−1^ until an audible ‘pop’ or a 10% decrease in load occurred, indicating the maximum load to failure. Mice were then recovered, returned to vivarium and monitored for use in an unrelated study. The failure model generates partial ACL tears within the proximal third with no macropathology to the surrounding joint structures (Ahn et al., 2023). From the resulting load‐displacement curves generated from each failure, moderate and strenuous submaximal loads were defined as 30% and 60%, respectively, of the body weight adjusted median *in vivo* ACL maximum load to failure. After determining the prescribed loads for each sex, the right knee and ACL were fatigue loaded for 440 cycles at a rate of 0.75 mm s^−1^. Over the course of the fatiguing protocol, the load was programmed to routinely shift between moderate and strenuous loading. The loading protocol began with 20 cycles at 30% max ACL failure load followed by 10 cycles at 60% maximum ACL failure load, which was then repeated throughout the test. After training, mice were recovered with heat support, returned to vivarium and monitored until death.

From the load‐displacement data acquired during fatigue loading of each mouse, a custom MATLAB code (MathWorks Inc., Natick, MA, USA) was utilized to calculate knee hysteresis and stiffness beginning at the 51st cycle to ensure that the knee mechanics had stabilized (Fung et al., 2010). Hysteresis was quantified as the area between the loading and unloading curves, which is a measure of the knees viscous response (Maganaris & Paul, 2000). Knee stiffness was quantified as the average of slopes of the linear portion of the loading and unloading curves, which is a measure of anterior knee laxity (Loflin et al., 2023). Hysteresis and stiffness were calculated separately for the 30% and 60% load/unload curves.

### Histochemistry

At 24 and 72 h post‐fatigue, a subset of paired hindlimbs were processed for paraffin histology (*n* = 4 per sex per timepoint). Immediately following death, paired hindlimbs were removed and fixed in 4% paraformaldehyde for 48 h, followed by rinsing in distilled water. Tissue was then decalcified in 10% ethylenediamine tetraacetic acid at 4°C until decalcification was confirmed via radiographic assessment (∼10 days), followed by rinsing in distilled water. Finally, tissue was serially dehydrated through graded ethanol and embedded in paraffin. Paraffin‐embedded tissue blocks of each knee were sectioned sagittally at 5 µm and encompassed the entire expanse of the ACL. Following this, a subset of tissue sections from mice killed 24 h post‐injury were deparaffinized and incubated with a sulfo‐Cyanine3 fluorophore‐conjugated collagen hybridizing peptide (R‐CHP; 3Helix, Salt Laek City, UT, USA). The CHP is specific to denatured collagen of all types (Zitnay et al., 2017). Additionally, tissue sections from mice killed at both post‐injury timepoints were incubated with a cleaved caspase‐3 primary antibody and a biotinylated goat anti‐human IgG secondary antibody (Vectastain Elite ABC‐HRP Kit; Vector Laboratories, Newark, CA, USA) for detection of apoptotic cells within the ECM using 3,3′‐diaminobenzidine (ImmPACT DAB; Vector Laboratories). Stained images were acquired at 10× magnification under fluorescence (TRITC filter, 250 ms exposure, 1× gain) or brightfield using a motorized microscope (Eclipse Ni‐U; Nikon Corp., Tokyo, Japan) equipped with a 16.25‐MP monochrome camera (DS‐Qi2; Nikon Corp.). The percentage positive CHP and caspase‐3 area was quantified across the entire ACL and associated entheses. For CHP, fluorophore expression was quantified using ImageJ (National Institutes of Health, Bethesda, MD, USA) and a custom macro to calculate the percentage area of ACL tissue containing denatured collagen. For caspase‐3, quantification of DAB stain was performed using ImageJ via colour thresholding and the Immunohistochemistry Image Analysis Toolbox plug‐in (Shu et al., 2013), to calculate the percentage area of tissue containing apoptotic cells.

### RNA sequencing

At 24 and 72 h post‐fatigue, a subset of paired hindlimbs were processed for RNA sequencing (*n* = 6 per sex per timepoint). Paired hindlimbs were immediately removed following expiration, trimmed of excess muscle and submerged in RNALater (Thermo Fisher Scientific, Waltham, MA, USA) at room temperature for 7 days to halt RNase activity and facilitate ACL removal without significant RNA degradation. Following this, paired knees were microdissected using stereomicroscopy (S Apo; Leica, Wetzlar, Germany) to remove each ACL. Extracted tissue was then individually flash frozen in liquid nitrogen, and stored at –80°C. To acquire RNA, individual ACLs were disrupted using a high‐speed mechanical homogenizer (Model 150; Thermo Fisher Scientific) in lysis buffer (RLT; Qiagen, Hilden, Germany) and β‐mercaptoethanol (Sigma‐Aldrich, St Louis, MO, USA). Each specimen was then centrifuged, and the resulting supernatant was then processed for DNA digestion, RNA extraction and purification using a RNeasy Plus Micro Kit (Qiagen). Total RNA concentration and RNA integrity was quantified using a bioanalyzer (2100; Agilent Technologies, Santa Clara, CA, USA). All samples achieved an RNA integrity number of 7 or greater, and the mean RNA concentration was 1200 pg µL^−1^. Because of the low concentration, two fatigued or contralateral control samples were pooled for all six samples (three left and three right ACL pools) acquired for each sex and post‐injury timepoint, resulting in a total of 24 pools. Pools were then provided to the Indiana University School of Medicine Center for Medical Genomics for cDNA synthesis and library prep using an ultra‐low input RNA kit (Smart‐Seq v4; Takara Bio, Shiga, Japan) followed by sequencing via a NovaSeq X system (Illumina, San Diego, CA, USA). After sequencing the quality of raw read data for each sample was checked using FastQC. (Andrew, 2010) Next, raw reads were aligned to the B6 mouse genome using STAR (Dobin et al., 2012) and then checked using FastQC. Gene expression quantification was then performed using featureCounts (Liao et al., 2014) to acquire counts of aligned reads for each gene, which was then used for differential gene expression analyses using edgeR (Huang et al., 2009). To correct for false discovery rate (FDR, *q* values), *P* values were adjusted for multiple hypotheses testing using the Benjamini–Hochberg procedure. Significant differentially expressed genes (DEGs) were defined as having a *q* < 0.05 and a log2 fold change greater than 1, relative to contralateral controls. Significant DEGs were uploaded to the Gene Ontology (http://geneontology.org) knowledgebase for taxonomic classification of biological processes, molecular functions and cellular components that involve sets of genes present in the dataset (Ashburner et al., 2000; The Gene Ontology Consortium, 2023; Thomas et al., 2022). Additionally, all read counts per million for each gene and sample were uploaded into ReactomeGSA (Griss et al., 2020) to identify significant differentially expressed pathways and functional groupings of genes both shared and unique to female and male datasets at each timepoint, as well as between timepoints for each sex. Pathway analysis was conducted without interactors using the Pathway Analysis with Down‐Weighting of Overlapping Genes (PADOG) method, which gives more weight to genes that are gene‐set specific rather to genes found in multiple gene sets (Tarca et al., 2012). Analysis did not include interactors or disease pathways. Significant differentially expressed pathways were defined as having a *q* < 0.05, a log2 fold change greater than 1 and at least four mapped genes.

### Statistical analysis

All statistical analyses were performed using Minitab, version 20 (Minitab LLC, State College, PA, USA) and Prism, version 10 (GraphPad Software Inc., San Diego, CA, USA). ClustVis was utilized for principal component analysis of all DEGs across post‐injury timepoints and sex (Metsalu & Vilo, 2015). Normality of data was assessed using the Shapiro–Wilk test. To test for mechanical (knee hysteresis and stiffness) and histochemistry (denaturation and programmed cell death) differences between males and females at each post‐injury timepoint, two‐tailed, paired *t* tests were used. All ACL and knee mechanical measures were adjusted for body weight using linear regression. Adjusted data was then compared between sexes and timepoints via ANOVA, after adjusting for body weight via linear regression. Data are reported as the mean ± SD. An alpha of 0.05 was considered statistically significant.

## Results

### ACL and knee mechanical properties are sex‐specific

**Figure 2:**
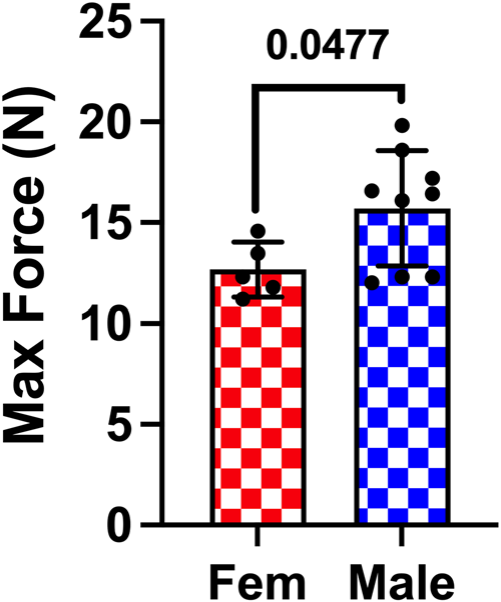
Female mice have weaker ACLs than those of males Body weight adjusted mean and standard deviations for *in vivo* ACL strength. *n* = 10 mice per sex. Mean ± SD bodyweights are 20.15 ± 0.67 g for females and 25.85 ± 1.52 g for males.

**Figure 3:**
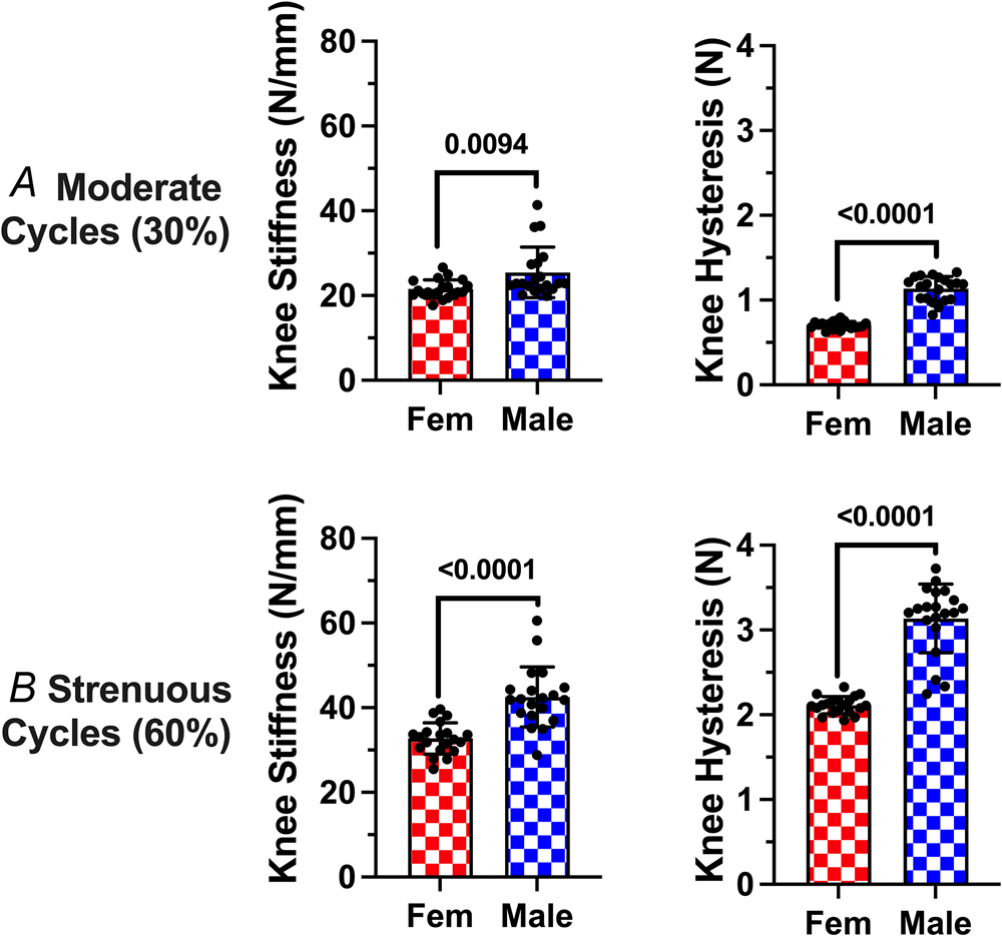
Female mice have less knee stiffness and hysteresis compared to males Body weight adjusted mean and standard deviations for knee stiffness and hysteresis at (*A*) moderate and (*B*) strenuous loads. *n* = 20 mice per sex. Mean ± SD bodyweights are 19.40 ± 1.03 g for females and 26.35 ± 1.52 g for males.

From the load to ACL failure tests used to delineate the moderate (30% maximum force) and strenuous (60% maximum force) loads, the resulting data confirmed a significant sex difference in *in vivo* ACL strength (*P* = 0.0477). Female ACLs had a mean failure load of 12.67 ± 1.36 N, whereas the mean failure load was 15.70 ± 2.87 N for male ACLs (Fig. 2). Additionally, from the loading curves generated throughout fatigue testing, there were significant sex differences in knee stiffness at both the moderate (*P* = 0.0094) and strenuous (*P* < 0.0001) loads (Fig. 3*A*). At both loads, female knees were less stiff compared to males. Mean female knee stiffness at the moderate and strenuous loads was 21.56 ± 2.28 and 32.76 ± 3.74 N mm^−1^, respectively, while mean male knee stiffness at both loads was 25.45 ± 4.48 and 42.53 ± 5.64 N mm^−1^, respectively. Furthermore, males demonstrated significantly greater knee hysteresis at both the moderate (*P* < 0.0001) and strenuous (*P* < 0.0001) loads compared to females (Fig. 3*B*). Moderate and strenuous loading in males demonstrated mean knee hysteresis of 1.14 ± 0.13 and 3.14 ± 0.39 N*mm, respectively, compared to a female knee hysteresis for each load of 0.70 ± 0.09 and 2.11 ± 0.18 N*mm, respectively. This hysteretic outcome is expected because the load to ACL failure, and thus the applied 30% and 60% maximum force loads, is higher in males compared to females.

### Significant sex differences in accrued denatured collagen but not apoptosis

**Figure 4:**
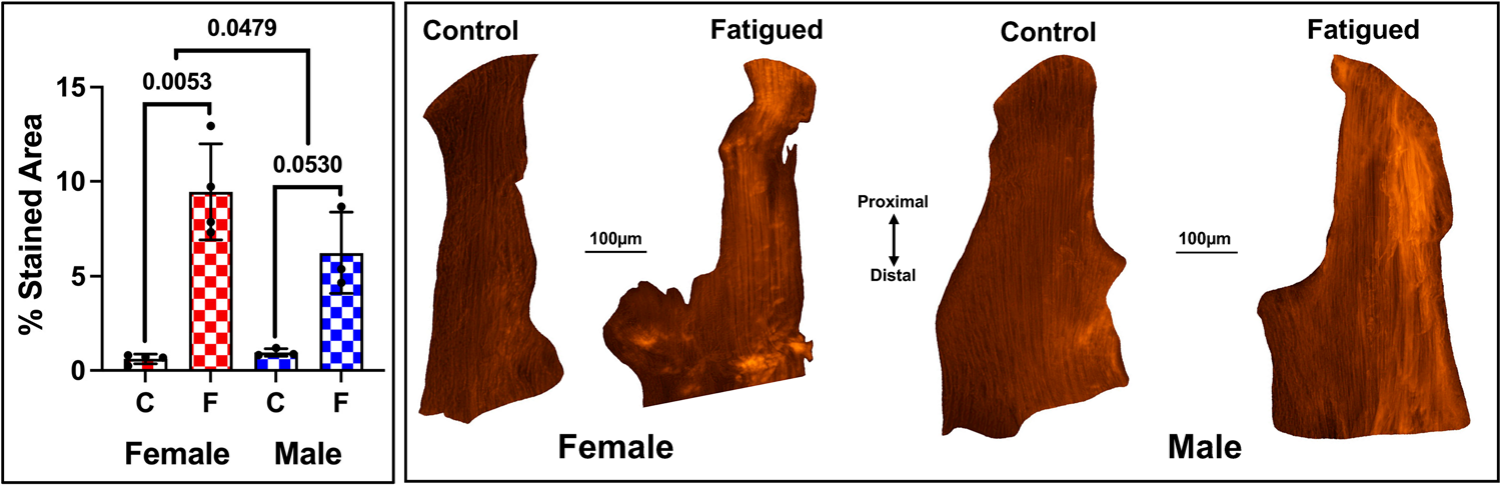
Female ACLs accrue more fatigue‐induced denatured collagen compared to males Left: mean percentage area of CHP‐detected denatured collagen at 24 h post‐injury. C = control ACL, F = fatigued ACL. *n* = 3–4 mice per sex. Each data point is the mean percentage across three to five tissue sections analyzed per knee. Right: paired male and female ACLs cropped from two mouse knees that represent the mean percentage area stained positive for denatured collagen.

Twenty‐four hours post‐injury both female and male ACLs showed a significant increase in the percentage area of denatured collagen post‐injury. There was a significant increase (*P* = 0.0288) in CHP‐detected denatured collagen in fatigued female ACLs (9.99 ± 2.83%) compared to their contralateral control ACLs (0.74 ± 0.07%). Similarly, males also showed a significant increase (*P = *0.0530) in denatured collagen in fatigued ACLs (6.23 ± 2.14%) compared to their contralateral control ACLs (0.96 ± 0.20%) (Fig. 4). Moreover, when fatigued female and male ACLs were adjusted to account for denatured collagen present in their paired control ACLs, females showed significantly more denatured collagen than males 24 h after fatigue loading (*P = *0.0479).

**Figure 5:**
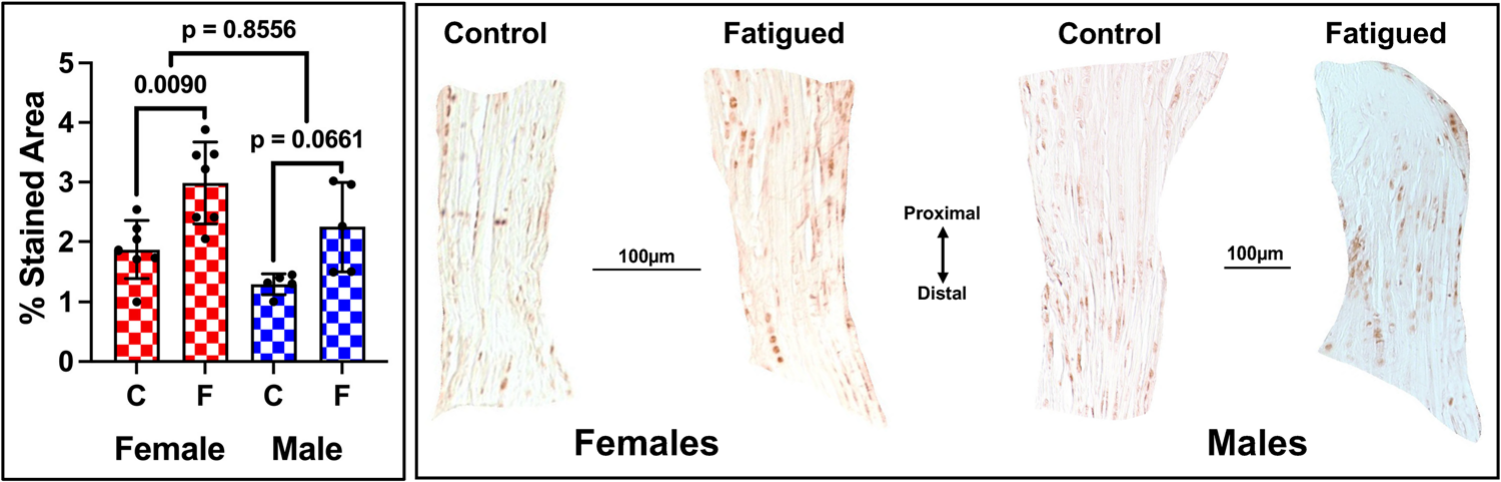
Fatigued female ACLs exhibit significant cell apoptosis compared to their non‐fatigued contralateral ACL Left: mean percentage area of Casp3‐detected apoptosis post‐injury. 24 h and 72 h are combined. C = control ACL, F = fatigued ACL. *n* = 6–7 mice per sex; Each data point is the mean percentage across four to six tissue sections per knee. Right: paired male and female ACLs cropped from two mouse knees that represent the mean percentage area stained positive for Casp3.

In terms of caspase‐3 (*Casp3*), both female and male fatigued ACLs demonstrated a significant percentage increase in apoptosis post‐injury. There was a significant percentage increase (*P* = 0.01) in *Casp3*‐detected apoptosis in fatigued female ACLs (2.98 ± 0.69) compared to their contralateral ACLs (1.87 ± 0.48). Similarly, fatigued male ACLs showed a higher percentage of *Casp3*‐detected apoptosis (2.25 ± 0.74) compared to their contralateral ACLs (1.29 ± 0.18), although this did not reach significance (*P = *0.07) (Fig. 5). When fatigued female and male ACLs were adjusted by their contralateral ACLs, there was no sex difference in the overall percentage of *Casp3* (*P = *0.86).

### Males demonstrate a greater number of significant upregulated and downregulated DEGs compared to females

**Figure 6:**
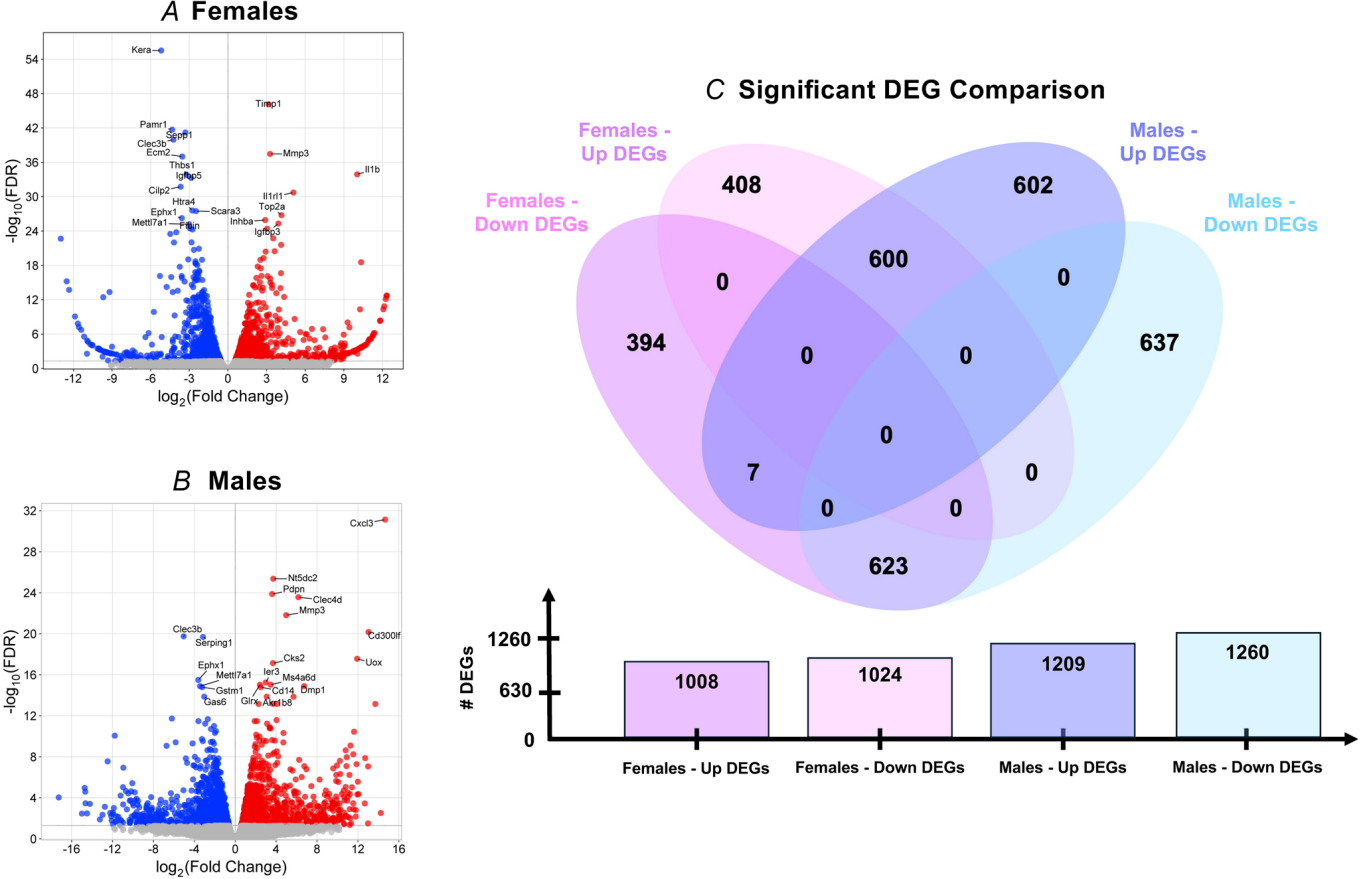
24‐h DEG comparisons Volcano plots by FDR and log2 fold change for (*A*) females and (*B*) males. *C*, venn diagram comparing significant (*q* < 0.05) upregulated and downregulated DEGs between females and males.

At 24 h post‐injury, females had 13,405 mapped genes, of which 2032 of these met the cutoff criteria for significant DEGs relative to contralateral controls (1008 upregulated and 1024 downregulated) (Fig. 6*A* and *C*). By contrast, males had 14,739 mapped genes, with 2469 of these being differentially expressed relative to contralateral controls (1209 upregulated and 1260 downregulated) (Fig. 6*B* and *C*). Top 10 upregulated and downregulated female and male DEGs by FDR are listed in Table 1.

**Table 1:** Top 10 upregulated and downregulated DEGs in ACLs 24‐ and 72‐h post‐injury.

Upregulated			Downregulated			Upregulated			Downregulated		
Gene	Log2FC	FDR	Gene	Log2FC	FDR	Gene	Log2FC	FDR	Gene	Log2FC	FDR
24 h Female DEGs						72 h Female DEGs					
*Col10a1*	5.55	8.94 × 10^−45^	*Lepr*	−3.83	7.08 × 10^−43^	*Timp1*	3.14	1.13 × 10^−50^	*Kera*	−5.18	3.03 × 10^−56^
*Cthrc1*	4.63	9.28 × 10^−41^	*Abi3bp*	−3.08	6.83 × 10^−38^	*Mmp3*	3.26	1.56 × 10^−41^	*Pamr1*	−4.32	2.04 × 10^−42^
*Col2a1*	2.99	5.24 × 10^−33^	*Angptl7*	−3.73	2.54 × 10^−36^	*Il1b*	10.02	7.61 × 10^−38^	*Sepp1*	−3.30	6.21 × 10^−42^
*Nt5dc2*	4.19	4.10 × 10^−24^	*Cilp*	−3.13	5.83 × 10^−35^	*Il1rl1*	5.08	1.71 × 10^−34^	*Clec3b*	−4.23	1.12 × 10^−40^
*Timp1*	2.69	5.36 × 10^−16^	*Clu*	−3.20	8.64 × 10^−35^	*Top2a*	4.15	1.92 × 10^−30^	*Ecm2*	−3.54	9.98 × 10^−38^
*Col6a3*	1.88	7.89 × 10^−15^	*Igfbp5*	−2.70	1.61 × 10^−29^	*Inhba*	2.89	1.54 × 10^−29^	*Thbs1*	−3.22	1.39 × 10^−34^
*Lyz2*	1.86	7.83 × 10^−14^	*Chad*	−2.86	1.06 × 10^−25^	*Igfbp3*	3.91	6.66 × 10^−29^	*Igfbp5*	−2.86	5.32 × 10^−34^
*Il1rl1*	4.39	2.71 × 10^−11^	*Cilp2*	−2.71	4.62 × 10^−25^	*Mcm5*	3.04	6.23 × 10^−28^	*Cilp2*	−3.67	1.84 × 10^−32^
*Postn*	3.20	8.95 × 10^−11^	*Fbln1*	−3.76	7.18 × 10^−25^	*Prc1*	4.12	5.71 × 10^−25^	*Htra4*	−2.76	2.60 × 10^−28^
*Stmn1*	3.38	1.55 × 10^−10^	*Cytl1*	−3.02	1.07 × 10^−24^	*Uhrf1*	3.62	8.13 × 10^−24^	*Scara3*	−2.48	3.67 × 10^−28^
24 h Male DEGs						72 h Male DEGs					
*Nt5dc2*	4.18	5.72 × 10^−32^	*Serping1*	−3.05	5.17 × 10^−19^	*Cxcl3*	14.65	7.31 × 10^−32^	*Clec3b*	−5.04	1.78 × 10^−20^
*Cks2*	4.10	4.53 × 10^−21^	*Clec3b*	−4.70	3.36 × 10^−18^	*Nt5dc2*	3.72	4.25 × 10^−26^	*Serping1*	−3.15	2.03 × 10^−20^
*Uox*	12.54	5.64 × 10^−20^	*Gstm1*	−2.85	2.93 × 10^−12^	*Pdpn*	3.61	1.33 × 10^−24^	*Ephx1*	−3.60	3.18 × 10^−16^
*Cxcl3*	11.05	5.17 × 10^−19^	*Gm7694*	−10.61	3.24 × 10^−11^	*Clec4d*	6.17	2.69 × 10^−24^	*Mettl7a1*	−3.43	1.28 × 10^−15^
*Cd300lf*	11.12	1.24 × 10^−15^	*Mettl7a1*	−2.74	2.58 × 10^−10^	*Mmp3*	4.98	1.53 × 10^−22^	*Gstm1*	−3.22	1.60 × 10^−15^
*Clec4d*	4.59	2.44 × 10^−15^	*Nrep*	−3.18	9.91 × 10^−10^	*Cd300lf*	13.03	6.75 × 10^−21^	*Gas6*	−3.03	1.34 × 10^−14^
*Tcf19*	3.82	6.28 × 10^−15^	*Klf9*	−2.07	2.90 × 10^−9^	*Cks2*	3.69	7.23 × 10^−18^	*Pamr1*	−6.19	1.83 × 10^−12^
*Cthrc1*	4.57	1.27 × 10^14^	*Gabarapl1*	−1.94	7.35 × 10^−9^	*Ier3*	2.98	5.59 × 10^−16^	*Fxyd1*	−2.68	2.09 × 10^−12^
*Rrm2*	5.51	1.65 × 10^−14^	*Rnf167*	−1.94	9.26 × 10^−9^	*Ms4a6d*	3.45	9.20 × 10^−16^	*Nrep*	−3.59	3.27 × 10^−12^
*Smc4*	2.23	3.74 × 10^−14^	*Ephx1*	−2.53	1.13 × 10^−8^	*Glrx*	2.41	9.28 × 10^−16^	*Fam124a*	−2.93	5.80 × 10^−12^

**Figure 7:**
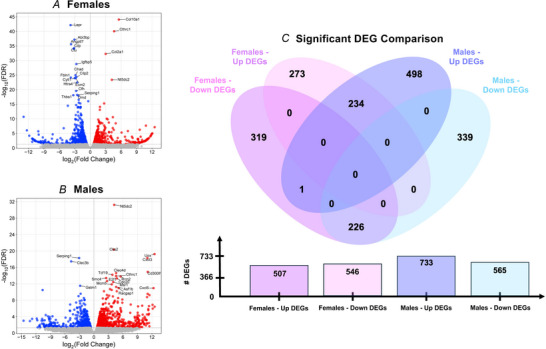
72‐h DEG comparisons Volcano plots by FDR and log2 fold change for (*A*) females and (*B*) males. *C*, venn diagram comparing significant (*q* < 0.05) upregulated and downregulated DEGs between females and males.

At 72 h post‐injury, females had 12,911 mapped genes with 1053 (507 upregulated and 546 downregulated) genes significantly differentially expressed (Fig. 7*A* and *C*). By contrast, males had 14,739 mapped genes with 1297 (733 upregulated and 565 down regulated) of these being significantly differentially expressed relative to contralateral controls. Top 10 upregulated and downregulated female and male DEGs by FDR are listed in Table 1. A list of all significant DEGs for males and females at 24 and 72 h post‐injury are provided in the Supporting information (File S1).

**Figure 8:**
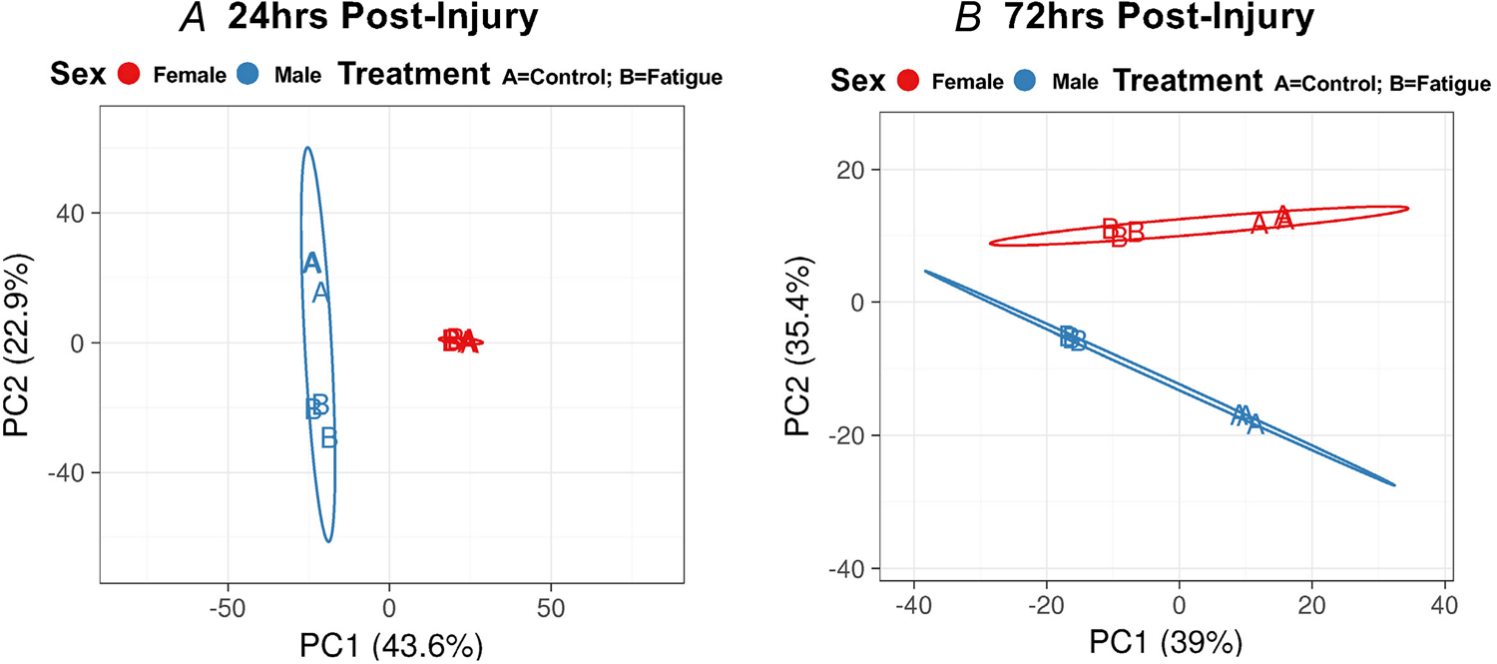
Principal component analysis Plots of PC1 and PC2 of log2 CPMs for male and female DEGs at (*A*) 24 h and (*B*) 72 h post‐injury.

Principal components analysis reinforced these gene expression differences between females and males. At 24 h post‐injury, female control and fatigued significant DEGs demonstrate little separation, as opposed to the males (Fig. 8*A*). Principal component 1 (PC1) explains 43.6% of the variance and PC2 explains 22.9% of the variance across female and male datasets. With the addition of PC3 (19%), the total variance explained from the top 3 components is 86%. By contrast, at 72 h post‐injury, both female and male fatigued datasets show clear separation from the control datasets (Fig. 8*B*). PC1 and PC2 explain 39% and 35.4% of the variance across female and male datasets, with the addition of PC3 explaining a total of 92% of the variance. All principal components and percentage variance explained at 24 and 72 h post‐injury are provided in Tables 2 and 3, respectively.

**Table 2:** Principal components and percentage variance explained by principal components for all significant DEGs shared by female and male ACLs 24 h post‐injury.

	PC1	PC2	PC3	PC4	PC5	PC6	PC7	PC8	PC9
**24 h Principal components**									
**Female control 1**	−24.73	0.92	−17.92	−0.48	−0.90	−2.96	0.61	8.02	−3.42
**Female control 2**	−24.00	0.25	−18.57	−0.23	−0.14	−5.97	−1.04	−7.00	−0.80
**Female control 3**	−24.87	0.01	−21.86	−0.22	−0.06	8.93	0.84	−1.10	3.17
**Female fatigue 1**	−20.50	1.13	19.04	−0.61	−0.32	−4.07	−0.52	2.20	7.61
**Female fatigue 2**	−18.91	0.69	20.40	−0.11	0.44	−1.02	−1.06	−1.82	−3.94
**Female fatigue 3**	−19.35	0.79	25.91	−0.28	0.35	5.03	1.48	−0.55	−2.78
**Male control 1**	24.20	24.56	−2.06	−7.01	5.08	0.55	0.20	0.01	−1.53
**Male control 2**	23.62	24.80	−1.68	−6.92	4.97	−0.41	−0.99	0.13	1.56
**Male control 3**	21.16	15.52	−0.63	20.90	−8.22	−0.13	1.26	−0.15	0.08
**Male fatigue 1**	23.55	−20.46	−0.30	−9.31	−8.08	−1.17	7.88	−0.70	0.20
**Male fatigue 2**	18.57	−29.23	−1.71	8.64	15.07	−0.30	0.31	0.35	0.14
**Male fatigue 3**	21.26	−18.97	−0.61	−4.37	−8.20	1.52	−8.97	0.61	−0.29
**Explained variance**									
**Individual**	0.44	0.23	0.19	0.05	0.04	0.01	0.01	0.01	0.01
**Cumulative**	0.44	0.66	0.86	0.91	0.95	0.96	0.97	0.98	0.99

**Table 3:** Principal components and percentage variance explained by principal components for all significant DEGs shared by female and male ACLs 72 h post‐injury.

	PC1	PC2	PC3	PC4	PC5	PC6	PC7	PC8	PC9
**72 h Principal components**									
**Female control 1**	−16.03	12.43	8.15	−2.39	−3.41	3.96	−1.41	0.10	−1.94
**Female control 2**	−15.64	13.00	7.91	−0.15	6.20	0.01	0.42	1.31	1.61
**Female control 3**	−12.08	11.80	4.38	2.22	−3.75	−4.92	1.43	−1.11	0.80
**Female fatigue 1**	10.38	11.04	−13.32	−6.73	0.11	−1.18	0.20	−0.78	0.84
**Female fatigue 2**	6.54	10.55	−8.81	3.69	2.39	0.21	−1.37	−3.38	−2.54
**Female fatigue 3**	9.10	9.80	−12.07	3.74	−1.66	2.02	0.83	3.88	1.09
**Male control 1**	−10.03	−17.04	−4.81	0.54	−0.89	1.16	−2.45	−1.80	3.81
**Male control 2**	−9.05	−16.91	−4.60	−0.27	0.68	−2.67	−2.96	2.50	−2.49
**Male control 3**	−11.53	−18.31	−5.38	−0.42	0.45	1.37	4.91	−0.64	−1.30
**Male fatigue 1**	16.30	−5.43	9.50	−1.41	−0.06	−1.67	0.40	1.45	−0.73
**Male fatigue 2**	15.19	−5.83	9.25	0.58	0.17	1.16	0.55	−1.64	−0.02
**Male fatigue 3**	16.86	−5.10	9.81	0.60	−0.22	0.56	−0.54	0.11	0.87
**Explained variance**									
**Individual**	0.39	0.35	0.18	0.02	0.01	0.01	0.01	0.01	0.01
**Cumulative**	0.39	0.74	0.92	0.94	0.95	0.96	0.97	0.98	0.99

### Ontology of gene function and location suggest sex differences in the response to injury

Gene Ontology analyses of the 24 and 72 h post‐injury datasets for females and males identified several over‐represented processes that were unique to one another. At 24 h, significant (*q < *0.05) female DEGs were skewed towards leukocyte migration, DNA replication, cell adhesion, migration and the cytoskeleton that were unique to their male counterparts. By contrast, male DEGs were uniquely over‐represented in catabolic processes and protein synthesis, in addition to strong cell adhesion processes (Table 4).

**Table 4:** Unique Gene Ontologies (GO) of significant DEGs for females and males 24 h post‐injury.

	GO	Terms	# Ref Genes	# Obs/Exp Genes	Fold	FDR
Unique 24 h female GOs						
**BP**	0000727	Double‐strand break repair	11	7/1	7.01	2.11 × 10^−2^
	0006271	DNA strand elongation	11	7/1	7.01	2.11 × 10^−2^
	0035335	Peptidyl‐tyrosine dephosphorylation	19	9/1.7	5.22	2.88 × 10^−2^
	0050900	Leukocyte migration	34	14/3.1	4.54	1.05 × 10^−3^
	0044772	Mitotic cell cycle phase transition	37	14/3.4	4.17	3.55 × 10^−3^
	051783	Regulation of nuclear division	33	12/3	4.01	3.08 × 10^−2^
	0071900	Regulation of serine/threonine kinase activity	54	16/5	3.27	2.63 × 10^−2^
	0006954	Inflammatory response	93	27/8.4	3.20	5.56 × 10^−5^
	0048285	Organelle fission	107	24/9.7	2.47	4.48 × 10^−2^
	2000026	Regulation of multicellular organismal development	142	31/12.9	2.41	8.11 × 10^−3^
	0051276	Chromosome organization	138	30/12.5	2.40	1.25 × 10^−2^
	0050793	Regulation of developmental process	251	46/22.8	2.02	6.89 × 10^−3^
	0051239	Regulation of multicellular organismal process	328	58/29.8	1.95	1.47 × 10^−3^
	0048870	Cell motility	301	53/27.3	1.94	4.34 × 10^−3^
	0051128	Regulation of cellular component organization	372	59/33.7	1.75	4.10 × 10^−2^
	1901135	Carbohydrate derivative metabolic process	457	69/41.5	1.66	4.22 × 10^−2^
	0006950	Response to stress	876	118/79.5	1.48	2.47 × 10^−2^
	1901564	Organonitrogen compound metabolism	2304	294/209	1.41	8.10 × 10^−7^
	0019538	Protein metabolic process	1803	215/163.6	1.31	3.85 × 10^−2^
**MF**	0017116	Single‐stranded DNA helicase activity	10	6/0.9	6.61	4.70 × 10^−2^
	0008094	ATP‐dependent activity, acting on DNA	50	15/4.5	3.31	1.29 × 10^−2^
	0005125	Cytokine activity	96	22/8.7	2.53	2.13 × 10^−2^
	0022836	* Gated channel activity *	238	5/21.6	0.23	1.10 × 10^−2^
**CC**	0005657	Replication fork	23	9/2.1	4.31	4.44 × 10^−2^
	0005874	Microtubule	148	29/13.4	2.16	2.97 × 10^−2^
	0099513	Polymeric cytoskeletal fibre	242	45/21.9	2.05	1.82 × 10^−3^
	0005929	* Cilium *	256	7/23.2	0.3	4.39 × 10^−2^
	0034702	* Monoatomic ion channel complex *	174	2/15.8	0.13	1.08 × 10^−2^
	1902495	* Transmembrane transporter complex *	220	2/20	0.1	1.58 × 10^−4^
Unique 24 h male Gos						
**BP**	0010811	Positive regulation of cell‐substrate adhesion	9	7/1	7.22	1.16 × 10^−2^
	0006270	DNA replication initiation	17	9/1.6	4.91	2.72 × 10^−4^
	0042273	Ribosomal large subunit biogenesis	47	18/5.1	3.55	3.53 × 10^−2^
	0007160	Cell‐matrix adhesion	43	15/4.6	3.24	2.22 × 10^−2^
	0072329	Monocarboxylic acid catabolic process	47	16/5.1	3.16	1.69 × 10^−2^
	0051241	Negative regulation of multicellular organismal process	53	17/5.7	2.98	7.68 × 10^−11^
	0044242	Cellular lipid catabolic process	89	25/9.6	2.61	2.93 × 10^−2^
	0046395	Carboxylic acid catabolic process	94	26/10.1	2.57	1.83 × 10^−3^
	0044282	Small molecule catabolic process	120	31/12.9	2.40	2.98 × 10^−2^
	0007015	Actin filament organization	148	34/15.9	2.13	7.92 × 10^−3^
	0022613	Ribonucleoprotein complex biogenesis	213	48/22.9	2.09	3.15 × 10^−7^
	0071495	cellular response to endogenous stimulus	212	46/22.8	2.01	2.75 × 10^−5^
	0051336	Regulation of hydrolase activity	240	51/25.9	1.97	1.88 × 10^−5^
	0044248	Cellular catabolic process	428	86/46.1	1.86	1.43 × 10^−4^
	0035556	Intracellular signal transduction	463	82/49.9	1.64	0.00 × 10^+0^
	0065009	Regulation of molecular function	515	87/55.5	1.57	9.24 × 10^−4^
	0031325	Positive regulation of cellular metabolic process	581	97/62.6	1.55	1.60 × 10^−4^
	0002443	* Leukocyte mediated immunity *	196	2/21.1	0.09	8.12 × 10^−21^
**MF**	0003735	Structural constituent of ribosome	108	26/11.6	2.23	3.72 × 10^−2^
	0097367	Carbohydrate derivative binding	275	58/29.6	1.96	3.44 × 10^−4^
	0140678	Molecular function inhibitor activity	181	38/19.5	1.95	2.96 × 10^−2^
	1901265	Nucleoside phosphate binding	262	53/28.2	1.88	4.14 × 10^−3^
	1901363	Heterocyclic compound binding	287	57/30.9	1.84	2.77 × 10^−3^
	0043168	Anion binding	353	67/38	1.76	2.04 × 10^−3^
	0016787	Hydrolase activity	1436	205/154.7	1.32	1.06 × 10^−2^
**CC**	0005832	Chaperonin‐containing T‐complex	8	6/0.9	6.96	1.55 × 10^−2^
	0000940	Outer kinetochore	9	6/1	6.19	4.23 × 10^−2^
	0022626	Cytosolic ribosome	79	28/8.5	3.29	2.29 × 10^−6^
	1990904	Ribonucleoprotein complex	418	73/45	1.62	1.32 × 10^−2^

Column 1 (C1) taxonomic classes are BP = biological process, MF = molecular function, CC = cellular component. C2 comprises gene ontology identifiers. C3 comprises terms. C4 comprises total number of reference genes in term. C5 comprises the number of genes observed in the dataset and number expected. C6 is the fold enrichment score and C7 comprises the false discovery rate calculated via Benjamin–Hochberg multiple comparisons test.

At 72 h, male DEGs continued to favor DNA replication and mitotic processes. Interestingly, there were no unique immune signalling processes significantly over‐represented. On the other hand, female DEGs continued to favor cytokine‐signalling, along with strong proteolysis regulation and muscle cell development. Both sexes were over‐represented in actin assembly, cell adhesion and ECM functions, but female processes appear to be coupled with muscle development, whereas males couple it with cell cycle regulation (Table 5). Full Gene Ontology lists for both sexes and post‐injury timepoints provided in the Supporting information (File S2).

**Table 5:** Unique Gene Ontologies (GO) of significant DEGs for females and males 72 h post‐injury.

	GO	Terms	# Ref Genes	# Obs/Exp Genes	Fold	FDR
Unique 72 h female Gos						
**BP**	0030239	Myofibril assembly	28	14/1.3	10.58	9.72 × 10^−9^
	0010927	Cellular component assembly	33	14/1.6	8.98	1.59 × 10^−7^
	0051146	Striated muscle cell differentiation	39	15/1.8	8.14	1.82 × 10^−7^
	0031032	Actomyosin structure organization	60	15/2.8	5.29	1.51 × 10^−4^
	0010951	Negative regulation of endopeptidase activity	62	14/2.9	4.78	1.56 × 10^−3^
	0010466	Negative regulation of peptidase activity	64	14/3	4.63	2.36 × 10^−3^
	0045861	Negative regulation of proteolysis	69	15/3.3	4.60	1.08 × 10^−3^
	0051346	Negative regulation of hydrolase activity	69	14/3.3	4.29	6.11 × 10^−3^
	0048646	Anatomical structure formation	109	21/5.1	4.08	6.12 × 10^−5^
	0140694	Organelle assembly	121	23/5.7	4.02	1.80 × 10^−5^
	0019221	Cytokine‐mediated signalling	100	17/4.7	3.60	7.44 × 10^−3^
	0034097	Response to cytokine	149	24/7	3.41	2.41 × 10^−4^
	0007015	Actin filament organization	148	21/7	2.98	4.26 × 10^−4^
	0032989	Cellular anatomical entity	199	28/9.4	2.60	2.89 × 10^−4^
	0097435	Supramolecular fibre organization	293	36/13.9	0.42	3.27 × 10^−2^
	0090304	* Nucleic acid metabolic process *	902	18/42.6	0.12	2.88 × 10^−2^
	0051606	* Detection of stimulus *	346	2/16.3	0.12	4.32 × 10^−5^
	0007600	* Sensory perception *	538	3/25.4	0.12	4.32 × 10^−5^
**MF**	0005201	ECM constituent	54	15/2.5	5.88	1.01 × 10^−5^
	0019838	Growth factor binding	44	10/2.1	4.81	1.70 × 10^−2^
	0003735	Structural constituent of ribosome	108	20/5.1	3.92	8.07 × 10^−5^
	0043167	* Ion binding *	619	52/29.2	1.78	3.77 × 10^−2^
**CC**	0022627	Cytosolic small ribosomal subunit	30	12/1.4	8.46	1.98 × 10^−6^
	0043292	Contractile fibre	76	22/3.6	6.12	1.52 × 10^−9^
Unique 72 h Male Gos						
**BP**	0007076	Mitotic chromosome condensation	6	5/0.3	14.57	6.15 × 10^−3^
	0000727	Double‐strand break repair	11	6/0.6	9.54	2.21 × 10^−2^
	0006270	DNA replication initiation	17	7/1	7.20	4.09 × 10^−2^
	0000070	Mitotic sister chromatid segregation	41	15/2.3	6.40	5.80 × 10^−6^
	0000079	Regulation of serine/threonine kinase activity	25	9/1.4	6.29	1.00 × 10^−2^
	0045785	Positive regulation of cell adhesion	28	10/1.6	6.24	3.26 × 10^−3^
	0098813	Nuclear chromosome segregation	62	18/3.5	5.08	1.13 × 10^−5^
	0007052	Mitotic spindle organization	35	10/2	5.00	3.16 × 10^−2^
	0006261	DNA‐templated DNA replication	61	16/3.5	4.59	3.72 × 10^−4^
	1901990	Regulation of mitotic cell cycle phase transition	63	16/3.6	4.44	6.03 × 10^−4^
	0000280	Nuclear division	93	21/5.3	3.95	8.53 × 10^−5^
	0048285	Organelle fission	107	22/6.1	3.59	2.45 × 10^−4^
	0001932	Regulation of protein phosphorylation	150	23/8.6	2.68	2.58 × 10^−2^
	0016477	Cell migration	246	34/14.1	2.42	3.86 × 10^−3^
	0048870	Cell motility	301	37/17.2	2.15	2.35 × 10^−2^
	0051128	Regulation of cellular component organization	372	43/21.3	2.02	2.28 × 10^−2^
	0003008	* System process *	658	14/37.6	0.37	1.41 × 10^−2^
**MF**	0005200	Structural constituent of cytoskeleton	36	10/2.1	4.86	1.31 × 10^−2^
	0019887	Protein kinase regulator activity	89	17/5.1	3.34	5.67 × 10^−3^
	0097367	Carbohydrate derivative binding	275	38/15.7	2.42	2.87 × 10^−4^
	0030234	Enzyme regulator activity	556	59/31.8	1.86	2.55 × 10^−3^
	0044877	Protein‐containing complex binding	582	60/33.3	1.8	6.17 × 10^−3^
	0003823	* Antigen binding *	227	1/13	0.08	2.14 × 10^−2^
**CC**	0000940	Outer kinetochore	9	5/0.5	9.71	2.72 × 10^−2^
	0005657	Replication fork	23	8/1.3	6.08	1.10 × 10^−2^
	0000779	Condensed chromosome, centromeric	47	15/2.7	5.58	1.21 × 10^−5^
	1902554	Serine/threonine kinase complex	65	14/3.7	3.77	6.30 × 10^−3^
	0005819	Spindle	76	16/4.3	3.68	2.15 × 10^−3^
	1902911	Protein kinase complex	74	15/4.2	3.54	6.85 × 10^−3^
	0015630	Microtubule cytoskeleton	446	49/25.5	1.92	5.04 × 10^−3^

Column 1 (C1) taxonomic classes are BP = biological process, MF = molecular function, CC = cellular component. C2 comprises gene ontology identifiers. C3 comprises terms. C4 comprises total number of reference genes in term. C5 is the number of genes observed in the dataset and number expected. C6 comprises the fold enrichment score and C7 is the false discovery rate calculated via Benjamin–Hochberg multiple comparisons test.

### Biomolecular pathways predicted by Reactome differ by sex across post‐injury timepoints

**Figure 9:**
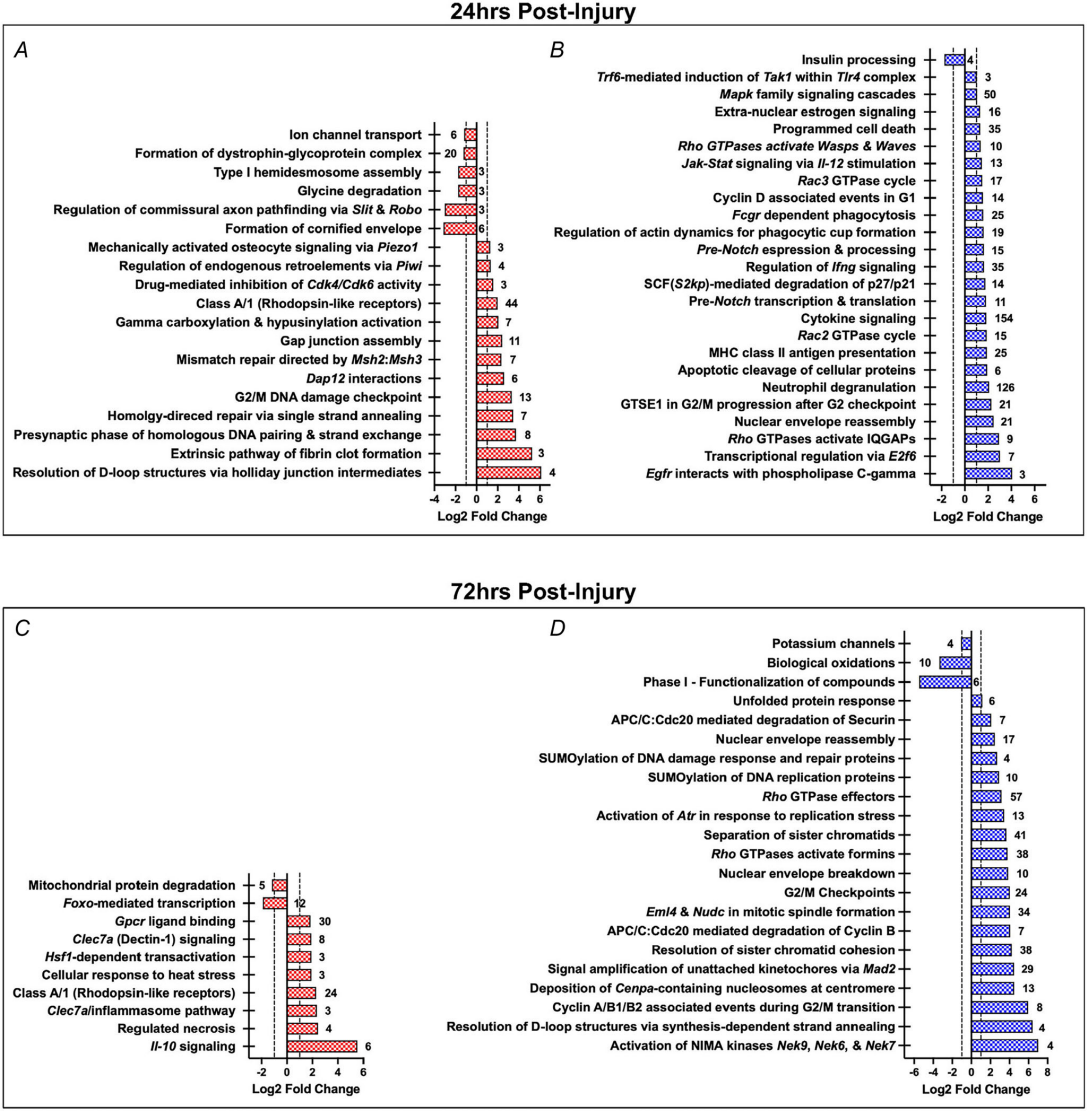
Unique enriched biomolecular pathways Unique enriched biomolecular pathways with number of associated genes (≥ 3 genes; *q* < 0.05; log2 fold change > 1). *A*, females 24 h post‐injury. *B*, males 24 h post‐injury. *C*, females 72 h post‐injury. *D*, males 72 h post‐injury.

Pathway analysis of significant (*P* < 0.05) female DEGs 24 h post‐injury yielded 55 enriched biomolecular processes that were predicted from at least 3 genes (Fig. 9*A*). Enriched pathways upregulated among females (*n* = 42) are involved in repairing DNA, modulating the immune response, growth factor signalling and mRNA metabolism. Downregulated female pathways (*n* = 13) are related to ECM/cytoskeleton anchorage, keratinization and mechanosignaling. In males, 110 significantly enriched pathways were identified 24 h post‐injury (Fig. 9*B*). Enriched pathways upregulated among males (*n* = 107) are involved in immune response activation, regulating apoptosis, growth factor signalling and RNA processing. Downregulated male pathways (*n* = 3) are related to insulin processing and transcription.

At 72 h post‐injury, 24 significantly enriched female pathways were identified (Fig. 9*C*). Enriched upregulated pathways among females (*n* = 17) are involved in the immune response, heat stress and ECM remodelling. Downregulated female pathways (*n* = 7) are related to metabolism and immune signalling. In males, 55 enriched pathways were identified (Fig. 9*D*). Enriched pathways upregulated in males (*n* = 43) are related to cell cycle regulation, responding to DNA damage and *Rho* GTPase signalling.

Downregulated male pathways (*n* = 12) are involved in neural function and metabolism. All significantly enriched female and male pathways containing at least 3 DEGs at 24‐ and 72‐h post‐injury are provided in the Supporting information (File S3).

## Discussion

Similar to humans, adolescent B6 female mice demonstrated up to 23% lower anterior knee stiffness (i.e. greater laxity) under load compared to males (Shultz et al., 2008). This outcome also mirrors reporting by others showing an *ex vivo* female bias towards greater anterior tibial displacement in young adult B6 mice (Liu et al., 2023). Females also had 24% weaker ACLs, compared to males, meaning less resistance to anterior tibial translation under multi‐axial forces (Butler et al., 1980). Part of our hypothesis was that this increased female knee laxity would result in greater fatigue‐induced ACL matrix damage, relative to males. This hypothesis was confirmed. Collagen denaturation among fatigued female ACLs was 43% more than that among fatigued male ACLs and was largely concentrated within the anterior proximal third of the ACL, as we have reported previously (Loflin et al., 2023). Both females and males showed increased apoptotic activity in response to ACL overuse; however, a significant sex difference was not observed.

Greater accrual of fatigue‐induced collagen matrix damage among female ACLs was further reflected in their downregulation of extracellular matrix genes (*Kera*, *Cilp2*, *Thbs1* and *Ecm2*). We also hypothesized a delayed or prolonged, physiological response to these collagen disruptions, relative to male ACLs. Although the outcomes of this study cannot definitively confirm or reject this part of the hypothesis, there is evidence to support a sex‐based difference in the initial physiological response to ACL overuse. At 24 h post‐injury, enriched pathways from female ACLs emphasize DNA repair and immune modulation. Featured DNA repair mechanisms and top differentially expressed genes (*Top2a*, *Mcm5*, *Prc1* and *Uhrf1*) indicate homologous recombination and the maintenance and/or restoration of genomic integrity during cell division. In response, females are also focused on regulating cell proliferation via clathrin‐mediated endocytosis. This pathway modulates proliferative activity by recycling endothelial growth factor receptors (*Egfr*) to prevent overaction of cell proliferative downstream signals. Concurrently, immune modulation is manifested through the signalling of *Il*‐*10*, a potent anti‐inflammatory cytokine, and is probably antagonizing the strong upregulation of *Il‐1b* in conjunction with an anti‐inflammatory interleukin receptor, *Il‐1rl1* (Dinarello, 2011). *Il‐10* can also assist in the regulation of *Dap12*, an adaptor protein, and rhodopsin‐like receptors involved in signalling immune cell activation for the clearance of cellular debris and pathogens in damaged tissue regions (Sun & Ye, 2012; Turnbull & Colonna, 2007). Further regulation of both cell proliferative activities and the inflammatory response is probably being provided via oestrogen receptor signalling. Oestrogen signalling can assist in reducing inflammation via inhibition of nuclear factor‐κB signalling (Liu et al., 2017; Stein & Yang, 1995), although potentially at the detriment of cell proliferation because there is some evidence suggesting a progressive decrease in ACL fibroblast proliferation with oestrogen exposure (Liu et al., 1997; Yu et al., 1999).

Male pathways at 24 h post‐injury indicate a dynamic environment consisting of immune activation, cytoskeletal organization, cell cycle regulation and hormonal signalling. Similar to females, males show significant upregulation of *Il‐10* to modulate their strong immune response, in part driven by *Cxcl3*, *Clec4d*, and *Cd300lf* pro‐inflammatory genes. Upregulated pathways involved in the male immune response include neutrophil degranulation, antigen recognition and interferon signalling, which are all involved in the removal of cellular debris and cellular stress defense mechanisms (Ethuin et al., 2004). These processes are co‐ordinated by cytokines and probably facilitated by cytoskeletal reorganization pathways involved in cell migration and phagocytosis. This is evident with the upregulation of actin dynamics, Fc gamma receptor (*Fcgr*) driven phagocytosis and *Rho* GTPases to enable immune cells to engulf and clear damaged cells and pathogens. To enhance and regulate the immune response, *Jak‐Stat* and *Mapk* signalling cascades are enriched. Further support for immune cell proliferation and survival is provided by the upregulation of the *Gab1 s*ignalosome and *Egfr* signalling, which, as for females, appears to be facilitated by clathrin‐mediated endocytosis. Males also show significant upregulation of oestrogen‐dependent signalling potentially to modulate the inflammatory response in a similar fashion to that of females. However, males have significantly less circulating oestrogen and immune cell oestrogen receptors compared to females, probably resulting in a more limited effect on resolving the inflammatory response (Hutson et al., 2019).

At 72 h post‐injury, females continue to prioritize modulation of the immune response by balancing anti‐inflammatory *Il‐10* and *Il‐1rl1* signalling and pro‐inflammatory *Clec7a* and *Lyz2* signalling. This uptick in inflammation is probably a direct result of regulated necrosis, which involves the lysing of damaged cells and the release of damage‐associated molecular patterns (i.e. DAMPs) (Murao et al., 2021). Upregulated pathways also indicate continued cellular stress with the activation of the heat shock response by *Hsf1*, and the arrest of cell cycle progression via the inhibition of proteolytic activity by the anaphase‐promoting complex (APC/C), thus preventing premature mitotic progression. Additional pathways suggestive of further immune modulation and cellular communication revolve around the upregulation of G‐coupled protein receptors (*Gpcrs*), such as rhodopsin‐like receptors, and ligand binding. There also may be an attempt to repair damage fibrocartilage within the proximal ACL enthesis, where the majority of denatured collagen was observed, with the upregulation of key cartilage matrix genes *Col10a1* and *Col2a1*. However, *Cytl1*, a regulator of chondrogenic differentiation of mesenchymal cells (Kim et al., 2007), is downregulated and there were no upregulated ECM remodelling pathways that were enriched.

By contrast to females, males 72 h post‐injury prioritize mitotic activities and rapid cell proliferation with the significant upregulation of *Nt5dc2*, *Cks2*, *Rrm2*, *Tcf19* and *Smc4*, along with enriched pathways involved in mitotic spindle formation, chromosome segregation, nuclear envelope breakdown and reassembly, and cyclin A, B1 and B2 events during the G2/M transition. Together, these processes indicate strong cell division activity and are further supported by APC/C:*Cdc20*‐mediated degradation of cyclin B, triggering the exit from mitosis. Males are not only emphasizing cell proliferation, but also cell migration with the upregulation of *Rho* GTPase formins that nucleate actin filaments essential for keratinocyte, fibroblast and endothelial cell movements, and *Rho* GTPase effectors that co‐ordinate these processes. However, the heightened cell proliferation and migration probably resulted in DNA replication stress because both ataxia‐telangiectasia and Rad3‐related kinase (*Atr*), an activator of the DNA damage response, and protein SUMOylation, a post‐transcriptional modification that facilitates the repair of genomic lesions, are simultaneously upregulated. Moreover, there are also indicators of endoplasmic reticulum stress related to misfolded proteins with the activation of the unfolded protein response (Hetz, 2012). Collectively, replication and endoplasmic reticulum stress suggest robust cell proliferation and protein synthesis as cells adapt to stress conditions (e.g. hypoxia) at the repair sites.

Overall, an acute ACL overuse injury results in greater collagen matrix damage in females compared to males, even with the applied loads to ACL being proportional to the sex‐specific difference in ACL strength. This difference is probably an effect of higher circulating oestrogen levels in females, which has been associated with an increase in knee laxity, a decrease in ACL stiffness and a reduction in collagen cross‐links, and thus greater susceptibility for ACL injury (Lee et al., 2015; Shultz et al., 2005; Slauterbeck et al., 1999). Despite females experiencing greater tissue damage, the biological response to these collagen disruptions at 24 h post‐injury demonstrates a controlled reparative process that prioritizes the restoration of DNA integrity at the same time as minimizing inflammatory activity. This is similar to what occurs in female innate immunity across species where the recruitment of neutrophils and disproportionate cytokine production are strongly regulated to dampen inflammation and reduce collateral damage via neutrophil‐derived mediators (Aragon‐Vela et al., 2021; Scotland et al., 2011). On the other hand, males prioritize the activation of a robust immune response, heightened debris clearance and cell proliferation during this same timeframe. This suggests a more aggressive repair strategy at the risk of driving an excessive inflammatory reaction. As for females, this is similar to what others have reported on the male response to musculoskeletal tissue damage (Aragon‐Vela et al., 2021). By 72 h post‐injury, females continue to prioritize modulation of the immune response, particularly as they upregulate cell necrosis and begin activating inflammatory cascades via *Clec7a* to recruit immune cells and clear debris. By contrast, males largely shift away from the inflammatory response and emphasize reparative processes through enhanced cell proliferation, cytoskeletal reorganization and protein stability.

Taken as a whole, the sex differences in the early response to ACL overuse suggests females are methodically taking steps that support sustained and regulated healing, whereas males are taking a more aggressive approach to accelerate tissue repair. Both strategies come with potential consequences. The more methodical approach taken by females may ultimately result in a more stable reparative process, although it may take them longer to restore ACL structure and function. The more aggressive approach taken by males may enable a faster reparative process, but may increase the risk of excessive fibrotic activity and scar formation (Ashcroft & Mills, 2002). However, in the context of sport, these sex‐specific reparative strategies may in part explain the female–male disparity in ACL overuse injury and failure rates. If female ACLs accrue a greater volume of matrix damage and have a slower reparative response, then high‐intensity reloading of the ACL prior to resolution of the injury could lead to further accumulation and propagation of matrix damage across collagen length scales (i.e. triple helix, fibril and fibre) (Chen et al., 2019; Kim et al., 2022; Loflin et al., 2023; Putera et al., 2023). By contrast, a faster reparative response in male ACLs could facilitate a quicker reengagement of high‐intensity activity at the same time as mitigating the immediate risk of a recurrent submaximal injury. If confirmed, this would warrant greater consideration for sex‐specific training and recovery regimens in load management. There is a current focus on enhancing athletic performance by emphasizing active management of loads experienced during training and conditioning (Buser et al., 2025; Ferraz et al., 2025; Leupold et al., 2025; Zaremski et al., 2025). A similar approach could be critical in preventing ligament overuse and subsequent tissue failures. Implementation of sex‐specific training and recovery strategies following an overuse injury may be necessary for effective ligament repair and the mitigation of excessive ACL wear and tear (i.e. matrix damage) (Nyland, 2023; Nyland et al., 2022).

## Additional information

## Competing interests

EMW is Editor‐in‐Chief of *Sports Health: A Multidisciplinary Approach* peer‐reviewed journal and the Chair of the Clinical Advisory Board for ProTherapeutics. No other authors have anything to disclose.

## Author contributions

S.H.S. was responsible for conceptualization, data curation, investigation, formal analysis, funding acquisition, methodology, project administration, resources, software, supervision, validation, writing – original draft, writing – reviewing & editing. B.E.L. was responsible for conceptualization, data curation, investigation, formal analysis, software, supervision, validation, writing – reviewing & editing. A.R.C. was responsible for investigation, formal analysis, software, writing – reviewing & editing. R.H. was responsible for data curation, investigation, formal analysis, software, writing – reviewing & editing. S.S. was responsible for data curation, formal analysis, writing – reviewing & editing. E.M.W. was responsible for conceptualization, formal analysis, writing – original draft, writing – reviewing & editing. All authors approved the final version of the manuscript submitted for publication and agree to be accountable for all aspects of the work. All persons designated as authors qualify for authorship and all those who qualify are listed.

## Funding

Support for this study includes NIH/NIAMS funding (SHS, AR070903).

## Supporting information




Peer Review History

Supplemental File 1

Supplemental File 2

Supplemental File 3

## Data Availability

All mechanical, histological and molecular data supporting the study results are reported in the paper itself and the associated Supporting Information.
